# The occurrence of *Cryptosporidium* sp., and eggs of soil-transmitted helminths in market vegetables in the north of Iran 

**Published:** 2019

**Authors:** Ali Taghipour, Ehsan Javanmard, Ali Haghighi, Hamed Mirjalali, Mohammad Reza Zali

**Affiliations:** 1 *Department of Parasitology, Faculty of Medical Sciences, Tarbiat Modares University, Tehran, Iran*; 2 *Department of Parasitology and Mycology, School of Medicine, Shahid Beheshti University of Medical Sciences, Tehran, Iran*; 3 *Foodborne and Waterborne Diseases Research Center, Research Institute for Gastroenterology and Liver Diseases, Shahid Beheshti University of Medical Sciences, Tehran, Iran*; 4 *Gastroenterology and Liver Diseases Research Center, Research Institute for Gastroenterology and Liver Diseases, Shahid Beheshti University of Medical Sciences, Tehran, Iran *

**Keywords:** Iran, North of Iran, Vegetables, Parasitic contamination

## Abstract

**Aim:**

This study aimed to investigate the presence of oocyst of *Cryptosporidium* sp., and egg of soil-transmitted helminths in market vegetables in the north of Iran.

**Background:**

Fecal-oral transmission via consumption of contaminated food is the main route of transmission of intestinal parasites. Concerning the high risk of contamination of vegetable with intestinal parasites, raw consumption of crops can enhance the chance of transmission of intestinal parasites.

**Methods:**

In this study, we collected 34 pre-washed vegetable samples including spinach, mint, parsley, oregano, chives, savory, radish, coriander, basil and tarragon from local markets in Tonekabon City, North of Iran. All vegetable samples were washed using sterile PBS. Parasitological examinations, including direct examination and staining with Lugol’s iodine and modified Ziehl–Neelsen were performed on the pellet resulted from the washing process.

**Results:**

The findings showed that 14/34 (41.17%) of collected samples were contaminated with at least one parasite. Eggs of *Toxocara* sp., *Ascaris* sp., *Fasciola* sp., *Toxoascaris* *leonine*, *Trichuris* sp. and *Enterobius* together with free-living larvae, amoeba cyst, cyst of *Entamoeba* *coli* and oocyst of *Cryptosporidium* sp., were observed among the positive samples. Furthermore, statistical analysis indicated that there was no significant correlation between parasitic contamination of vegetables and seasonal changes.

**Conclusion:**

This study signifies that some parasites due to their resistant cell wall usually remain in an environment with the harsh condition and thus, consumption of raw vegetables increases the risk of transmission of them.

## Introduction

 Although there are significant differences in distribution and infecting-genus, intestinal parasites, including helminths and protozoans, are still reported from both developing and developed countries (1, 2). 

It is suggested that fecal-oral transmission via the consumption of raw foods polluted with stool is the main route of parasitic infections (3). Regarding the global water crisis, either traditional irrigation of farmland using raw wastewater and fertilizing with raw manure or night soil are still conventional in most countries (4, 5). The main reason that farmers prefer to use wastewater to irrigate their farms, is the limitation of water resources. Beside this limitation, industrial and human raw wastewater are inexpensive resources that are currently used not only for irrigation but also to fertilize the farmlands (5, 6). 

Notwithstanding, raw sewage brings pathogenic microbes, including bacteria, viruses, and parasites to vegetable farms. As a consequence of this phenomenon, the risk of contamination with enteric pathogens in downstream vegetable farms would be dramatically increased (7-12). 

Furthermore, concerning the rich source of vital components of vegetables, consumption of particularly raw vegetables is widely accepted, all over the world (13, 14). However, consumption of raw vegetables can increase the risk of transmission of parasites, particularly in those subjects who suffer from the insufficient immune system (15, 16). Several proven outbreaks of microsporidia (17), *Cryptosporidium*(18-20) and *Fasciola* (21, 22) due to consumption of either raw or ready-to-use vegetables and salads have highlighted the public health importance of the use of raw vegetables and fruits even in developed countries. In Iran, some studies showed a high prevalence of parasitic contamination in raw vegetables (23-25). Nevertheless, only one study reported soil-transmitted helminths from raw vegetables from north of Iran (26). Therefore, concerning climate conditions of northern provinces of Iran such as high humidity, presence of traditional wastewater system, suitable temperature as well as traditional cultivation and irrigation of vegetables in some regions, it seems that studies of parasitic contamination of ready-to-eat vegetables would be interesting. However, the current study aimed to investigate parasitic contamination of pre-washed vegetables collected from Tonekabon, north of Iran, regarding the season. 

## Methods

**Location**

**Figure 1:**
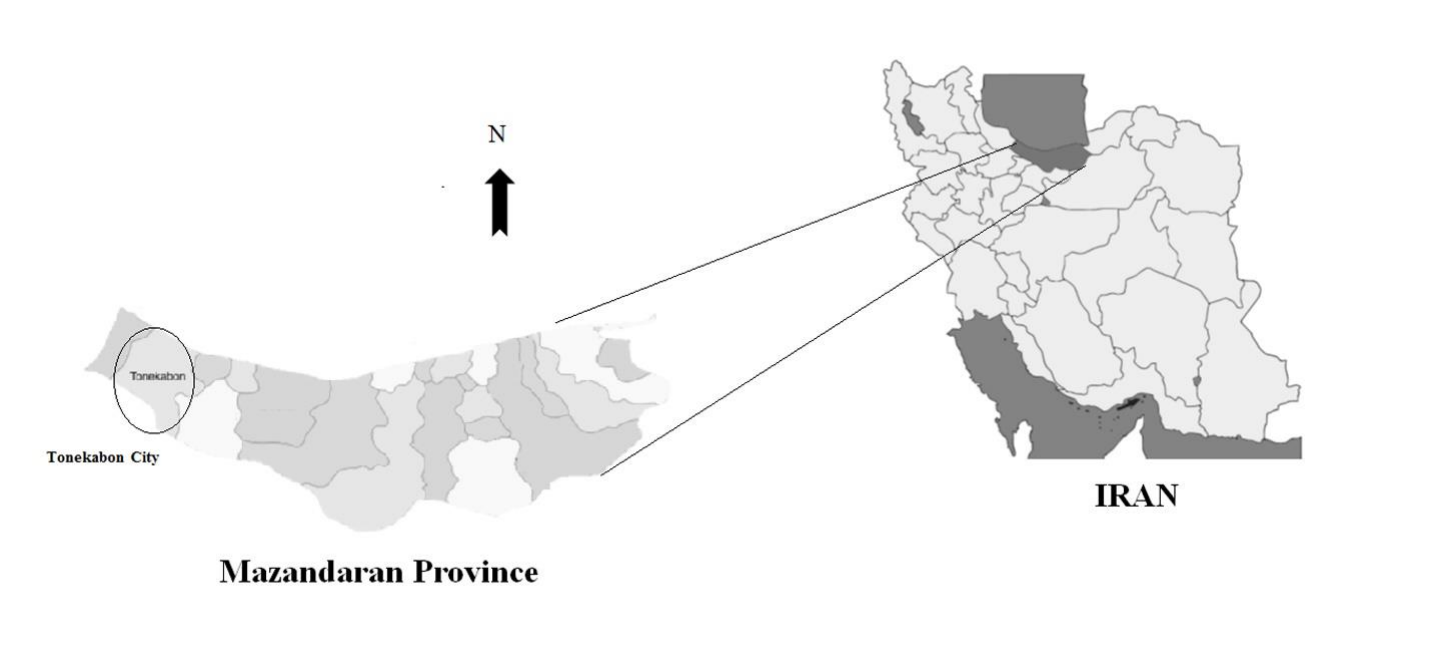
Map of Mazandaran province and location of Tonekabon city

This study was performed in Tonekabon city, Mazandaran province located in the north of Iran (Fig 1). The population of this city is estimated to be ~ 153,940, and the town has coastline with the Caspian Sea. In addition, the mean temperature of Tonekabon is 16 °C, and mean relative humidity value is 73%. The annual rainfall is between 1100 and 1500 mm. 

**Sample collection**

Fresh vegetable samples were randomly collected from central markets that brought fresh vegetables and fruits from different agricultural farms. Thirty-four pre-washed vegetable samples including spinach, mint, parsley, oregano, chives, savory, radish, coriander, basil and tarragon, were collected during a year (spring: 8; summer:8; autumn: 10 and winter: 8) from local markets. All samples were collected in sterile polyethylene bags and immediately transferred to the parasitology laboratory for further investigations. 

**Sample preparation and examination**

The samples were weighted in 250-gram portions and washed using 1L sterile PBS. All washed materials were transferred to a 2-liter graduated cylinder and kept out in the laboratory for 24h at room temperature to complete sedimentation of suspended materials. Then, the supernatant was discarded and remained sediment was centrifuged at 5000 rpm for 20 min. Finally, acquired pellets were stained using Lugol’s iodine and modified Ziehl-Neelsen and investigated for parasites under light microscopy (40X and 100X magnifications). In order to confirm the results of microscopical examinations and check the functionality of microscopic examination, positive slides for protozoa ((oo) cysts and trophozoites) and also ova of helminths were examined together with our slides. Indeed, all laboratory procedures in the present study were performed by well-trained researchers, and two expert parasitologists confirmed the positive samples.

**Statistical analysis**

Prevalence of parasites was calculated with a 95% confidence interval (CI). To assess the statistical association between the presence of parasites and seasonal change as well as the type of vegetables, the Fisher's Exact Test and Chi-square implemented in IBM SPSS Statistics for Windows, v22 (Chicago, IL, USA) were employed. A probability *P*-value <0.05 was considered statistically significant. 

## Results

The current findings showed that 14/34 (41.17%; 95% CI: 0.24-0.59) of collected samples were contaminated with either each of protozoan and helminth or both of them. Generally, protozoan parasite, helminth (larvae, egg) and mixed contamination were seen in 5/14 (35.71%; 95% CI: 0.35-0.14, P-value 0.62), 8/14(57.14%; 95% CI: 0.57-0.35, P-value 0.83) and 1/14 (7.14%; 95% CI: 0.07-0.00, P-value 0.33) of samples, respectively. *Toxocara* sp. eggs 3/14 (21.42%), *Ascaris* sp. egg 2/14 (14.3%), *Fasciola* sp. egg-like 1/14 (7.14%), Toxoascaris leonine egg 1/14 (7.14%), *Trichuris* sp. egg 1/14 (7.14%), *Enterobius* sp. egg 1/14 (7.14%), free-living larvae 3/14 (21.42%), amoeba cyst 3/14 (21.42%), cyst of *Entamoeba* *coli* 1/14 (7.14%), oocyst of *Cryptosporidium* 3/14 (21.42%) were observed among the positive samples (table 1). 

According to the seasonal changes, most of the parasitic contamination was seen in those samples that were gathered during spring (5/14) followed by autumn (4/14), winter (3/14) and summer (2/14). Fisher’s Exact Test showed that although most of the parasitic contaminations were seen in spring, there was no statistically significant correlation between seasonal change and presence the parasites (P-value=0.571). Moreover, the highest prevalence of parasitic contamination was observed in chives, but statistical analysis showed no significant correlation between the type of vegetable and the presence of parasite (P-value=0.694). Relationship between parasitic contamination and variety of vegetables regarding seasonal change was assessed, but with P-value=0.694, a statistically significant association was not seen.

## Discussion

The presence of parasites in food, particularly vegetables, is still considered one of the most critical public health issues. The main reason of this fact comes from changes in the lifestyle of communities that prefer to eat raw or slightly-cocked vegetables as well as contamination of vegetables during production, transport, preparation, and cocking. It is well-established that the consumption of raw vegetables significantly increases the chance of infection with particularly those microbes that can remain viable in harsh conditions (10, 27, 28). However, most of the parasites can remain infective, because of the presence of resistant stages such as cysts/oocyst and ova during their life. Several outbreaks due to the consumption of contaminated food have highlighted the pivotal role of parasites (17, 20, 29) in foodborne epidemies. Therefore, monitoring of parasitic contamination of foods, particularly vegetables, can provide a clear view of the potential risk of foodborne parasites in a region.

In the current study, parasites were seen in 14/34 (41.17%) of collected vegetables. Accordingly, eggs of *Tixocara* sp., *T. leonine*, *Ascaris* sp., *Enterobius* sp., *Trichuris* sp. and *Fasciola* sp. together with trophozoite/(oo)cysts of amoeba, *E. coli* and *Cryptosporidium *were the observed parasites. In this study, contamination with helminths was observed in 8/14 (57.14%) of vegetable samples. Interestingly, the prevalence of helminthic contamination in the current study was significantly higher than the previously published papers in Turkey, Sudan, and Nigeria. In a survey conducted by Adanir and Tasci in Turkey, the egg of helminths was seen in 6.3% of vegetable samples collected from bazaar (30). After that, Adenusi et al., observed only 8.44% of collected samples contaminated with helminthic eggs (31). Another study performed by Mohamed et al., 13.5% of vegetable samples were contaminated with intestinal parasites that the prevalence of helminthic eggs was significantly lower than protozoan parasites (32). Several studies in Iran also reported helminthic contamination of vegetables collected from markets. Ezatpour et al. observed helminthic eggs in samples collected in both spring and winter (25). In a study carried out by Rostami et al., soil-transmitted helminths were identified among 14.89% of vegetable samples that were collected from the north of Iran (26). However, the higher prevalence of helminthic eggs in our study in comparison with the mentioned study can be related to the sample size.

**Table 1:** Detected parasites in pre-washed vegetables collected from Tonekabon, regarding type of vegetable and season

Sampling time	Type of vegetable	Detected parasites	
		Helminth (larvae/eggs)	Protozoan ((oo)cyst/trophozoite)
Autumn	Spinach	*Toxocara* sp.	-
	Mint	-	-
	Parsley	-	-
	Oregano	Free-living larvae	-
	Chives	*Ascaris* sp.	-
	Savory	-	-
	Radish	-	-
	Coriander	-	-
	Basil	*Ascaris* sp.	-
	Tarragon		-
Winter	Mint	Free-living larvae *Fasciola* sp. *Toxocara* sp. *Trichuris* sp.	Amoeba cyst
	Parsley	-	-
	Chives	-	-
	Reddish	Free living larvae	-
	Basil	-	-
	Cress	*Enterobius* sp.	-
	Coriander	-	-
	Scallion	-	-
Spring	Chives	*Toxocara* sp.	-
	Basil	-	Amoeba cyst *Cryptosporidium* sp.
	Savory	-	*Cryptosporidium *sp*.*
	Coriander	-	-
	Reddish	-	-
	Parsley	-	*Entamoeba coli*
	Mint	-	-
	Cress	*Toxoasxaris* *leonine*	-
Summer	Scallion	-	-
	Mint	-	-
	Reddish	-	-
	Cress	-	-
	Coriander	-	*Cryptosporidium *sp.
	Chives	-	Amoeba cyst
	Basil	-	-
	Parsley	-	-

Notably, almost all of the detected helminth’s eggs in this study could be transmitted from non-human sources. Therefore, the presence of these helminth’s eggs in vegetables reflects either illegally use of manure or night soil to fertilize vegetable farms or free access of domesticated/wild animals to vegetable farms (33). Moreover, uncontrolled use of treated or untreated wastewater can enhance the risk of spreading parasites to vegetable farms. 

In this study, oocyst of *Cryptosporidium *was also detected among 3/14 (21.42%) of gathered vegetable samples. *Cryptosporidium *was reported from sold vegetables in southern Ethiopia (34).

Fallah et al., also reported *Cryptosporidium *from fresh salad vegetables in Shahrekord, west of Iran (23). *Cryptosporidium* is a zoonotic protozoan that is reported from both human and animals in which led to chronic diarrhea in immunocompromised patients (35). Several studies have indicated the presence of viable oocyst of *Cryptosporidium *in final effluent of wastewater treatment plant (36-38) due to their resistant wall (33). However, presence of resistant (oo)cyst/egg of parasites in vegetables sold in the local market indicates the public health importance of raw or slightly cooked vegetables, particularly, in susceptible groups such as children or individuals with compromised immunity (39).

The current study indicated that (oo)cyst and egg of potentially pathogenic parasites were seen in vegetables that were routinely sold in local markets. This study signifies the role of raw consumption of vegetables into the transmission of parasites that usually remain in the environment and harsh conditions due to their resistant cell wall.
